# Survival analysis of recurrent ovarian cancer under different PARP inhibitor treatment patterns: a single-center retrospective study

**DOI:** 10.3389/fonc.2024.1504084

**Published:** 2025-01-10

**Authors:** Jingtian Shen, Xi Wang, Olivier Mpano, Ying Wang, Yihan Shan, Xinning Lou, Piaopiao Ye, Xiaojian Yan

**Affiliations:** ^1^ Department of Gynecology, The First Affiliated Hospital of Wenzhou Medical University, Wenzhou, Zhejiang, China; ^2^ Department of Obstetrics and Gynecology, Taizhou Hospital of Zhejiang Province Affiliated to Wenzhou Medical University, Taizhou, Zhejiang, China

**Keywords:** ovarian cancer, PARP inhibitors, retrospective study, survival analysis, recurrence characteristics

## Abstract

**Objective:**

To compare the effects of different treatment modes containing PARPis and traditional treatment modes on the survival of patients with recurrent ovarian cancer.

**Methods:**

From December 2012 to December 2023, 131 recurrent ovarian cancer patients were screened. The patients were followed up retrospectively, and the relevant data was collected and analyzed.

**Results:**

Eighty-three patients used PARPis throughout the treatment process, and the median OS was not reached. Forty-eight patients did not use PARPis, and the median OS was 45.4 months. The two groups ‘ BRCA gene status, NACT, postoperative residual disease status, and PFI differ (*P* < 0.05). There was no significant difference in recurrence characteristics between the PARPis use and non-use groups in first-line maintenance therapy (*P* < 0.05). The use of PARPis, CA125 level and PFI were the independent influencing factors of OS in patients with recurrent ovarian cancer (*P* < 0.05). The median OS of patients with PARPis maintenance treatment in the single-line, second-line and last-line has not been reached. The median OS in the multi-line group was 69.5 months.

**Conclusion:**

The use of PARPis, CA125 level and PFI were independent influencing factors of OS in patients with recurrent ovarian cancer. The first-line maintenance use of PARPis will not cause differences in disease recurrence characteristics. Compared with the patients without PARPis, patients with recurrent ovarian cancer receiving PARPis maintenance therapy have longer OS. The group of patients with PARPis maintenance treatment in the second and last lines showed better OS (*P* < 0.05). However, OS was not significantly different between the second-line and last-line groups (*P* < 0.05). There was no significant difference in OS between the multiple-line use PARPis and single-line use PARPis groups.

## Introduction

Ovarian cancer is the second leading cause of death among gynecological cancers in China (1) and the United States (2). Due to its insidious onset and high recurrence rate, ovarian cancer poses a significant threat to women’s health globally (3). According to GLOBOCAN data (4), there were 324,398 new cases of ovarian cancer and 206,839 related deaths worldwide in 2022.

Standardized treatment of ovarian cancer includes surgery and chemotherapy (5). In the past decade, the introduction of the targeted drug poly (ADP-ribose) polymerase inhibitors (PARPis) have revolutionized the treatment of ovarian cancer, establishing a new paradigm that combines surgery, chemotherapy and maintenance therapy (6). PARP inhibitors have paved the way for precision medicine in ovarian cancer treatment (7). Alongside PARPis, targeted therapies such as vascular endothelial growth factor inhibitors (e.g., bevacizumab) (8) and BRAF V600E kinase inhibitors and MEK inhibitors (e.g., vemurafenib and cobimetinib) (9), as well as tumor biomarkers like breast cancer susceptibility gene (BRCA), RADiation sensitive protein 51 (RAD51), and Partner and Localizer of BCA2 (PALB2) (10), have emerged to meet the evolving needs of the field, offering expanded treatment opportunities for patients with ovarian cancer.

Currently, the Food and Drug Administration (FDA) has approved the use of three PARPis for the maintenance treatment of platinum-sensitive recurrent ovarian cancer patients: olaparib, niraparib, rucaparib and talazoparib (11). Due to significant differences in clinical trial design and patient populations, there is no direct evidence to indicate which PARPis has superior efficacy (12). The prevailing international consensus is that these three PARPis can significantly prolong PFS and chemotherapy intervals with comparable efficacy. However, there are notable differences in their safety profiles (13). Additionally, some studies have suggested that olaparib may demonstrate a more pronounced improvement in PFS in indirect comparisons (14).

As follow-up periods lengthen, several large-scale randomized controlled trials, such as PAOLA-1 (15), SOLO-1 (16), NORA (17), and BGB-290-102 (18), have demonstrated that PARPis can offer overall survival (OS) benefits, and its indications are continually expanding. However, these trials did not restrict the use of PARPis in the follow-up treatment of patients in the placebo group, leading to subsequent cross-use of PARPis among these patients. Therefore, the conclusions regarding PARPis efficacy require further validation in real-world clinical settings, comparing traditional treatment regimens with those that include PARPis.

Based on this background, we collected clinical data from patients diagnosed with recurrent ovarian cancer at the First Affiliated Hospital of Wenzhou Medical University between 2012 and 2023. Our study aims to analyze which baseline characteristics are affected by the use of PARPis and to determine whether PARPis influences the recurrence patterns. Additionally, we seek to verify the benefits of PARPis on OS using real-world clinical data and to identify independent factors influencing OS in ovarian cancer patients. Furthermore, we aim to investigate the impact of using PARPis as maintenance therapy at different stages on OS and to assess whether multiple courses of PARPis use can improve OS.

## Methods

### Study design and patient admission criteria

This single-center retrospective study collected clinical data from patients with recurrent epithelial ovarian cancer treated at the First Affiliated Hospital of Wenzhou Medical University between December 2012 and December 2023. Clinical data before relapse included age of diagnosis, BMI, CA125 level before initial treatment, BRCA gene status, FIGO staging, initial treatment date, neoadjuvant chemotherapy, postoperative residual disease status, pathological type, postoperative chemotherapy-related data, the efficacy of first-line platinum-containing chemotherapy and use of PARPis during first-line treatment. Clinical data after relapse included the date of initial recurrence, recurrence characteristics, initial treatment time after relapse, the second-line treatment plan, and the use of PARPis in the second-line and last-line treatments. The study was approved by ethics committee for clinical research at the First Affiliated Hospital of Wenzhou Medical University (KY2024-R224).

The inclusion criteria were as follows (1): Histologically confirmed epithelial ovarian cancer (2); Patients received surgery combined with platinum-containing chemotherapy as first-line treatment (3); The first recurrence occurred during the follow-up period, with evidence of tumor recurrence confirmed by imaging, pathology, or tumor markers. The exclusion criteria were as follows (1): Presence of other malignant tumors (2); Complications with hematological diseases, immune system diseases, severe liver or kidney dysfunction, or other serious medical conditions (3); Disease progression occurred during first-line platinum-containing chemotherapy or within one month after the last chemotherapy session (patients with primary platinum-refractory ovarian cancer) (4); Incomplete first-line treatment for ovarian cancer (including surgery and platinum-containing chemotherapy) (5); Incomplete second-line treatment following recurrence during the follow-up period (6); Loss to follow-up or lack of essential clinical data.

The primary clinical data of patients with initial recurrence were collected through electronic medical records. Recurrence and survival outcomes were obtained by reviewing outpatient and inpatient medical record systems and conducting telephone follow-ups, centralized in December 2023.

### Statistical analysis

Overall survival (OS) was defined as the time interval from the date of the first treatment to the time of death or the last follow-up.

Data were analyzed using SPSS 25.0 and MedCalc 18.2.1. The Shapiro-Wilk test was employed to assess the normality of measurement data. Data conforming to a normal distribution were presented as mean ± standard deviation and compared between groups using an independent samples t-test. For data that did not conform to a normal distribution, the median (25th to 75th percentile) was used for description, and the Mann-Whitney U test was applied for intergroup comparisons. Categorical data were described by frequency and percentage, with comparisons using Fisher’s exact test. A Cox regression model was used to analyze OS in patients with recurrent ovarian cancer through univariate analysis, and variables with *P* < 0.05 were included in a multivariate analysis. Survival curves were generated using the Kaplan-Meier method, and univariate analysis of OS was conducted with the log-rank test. In this study, *P* < 0.05 was considered statistically significant.

## Results

**Figure 1:**
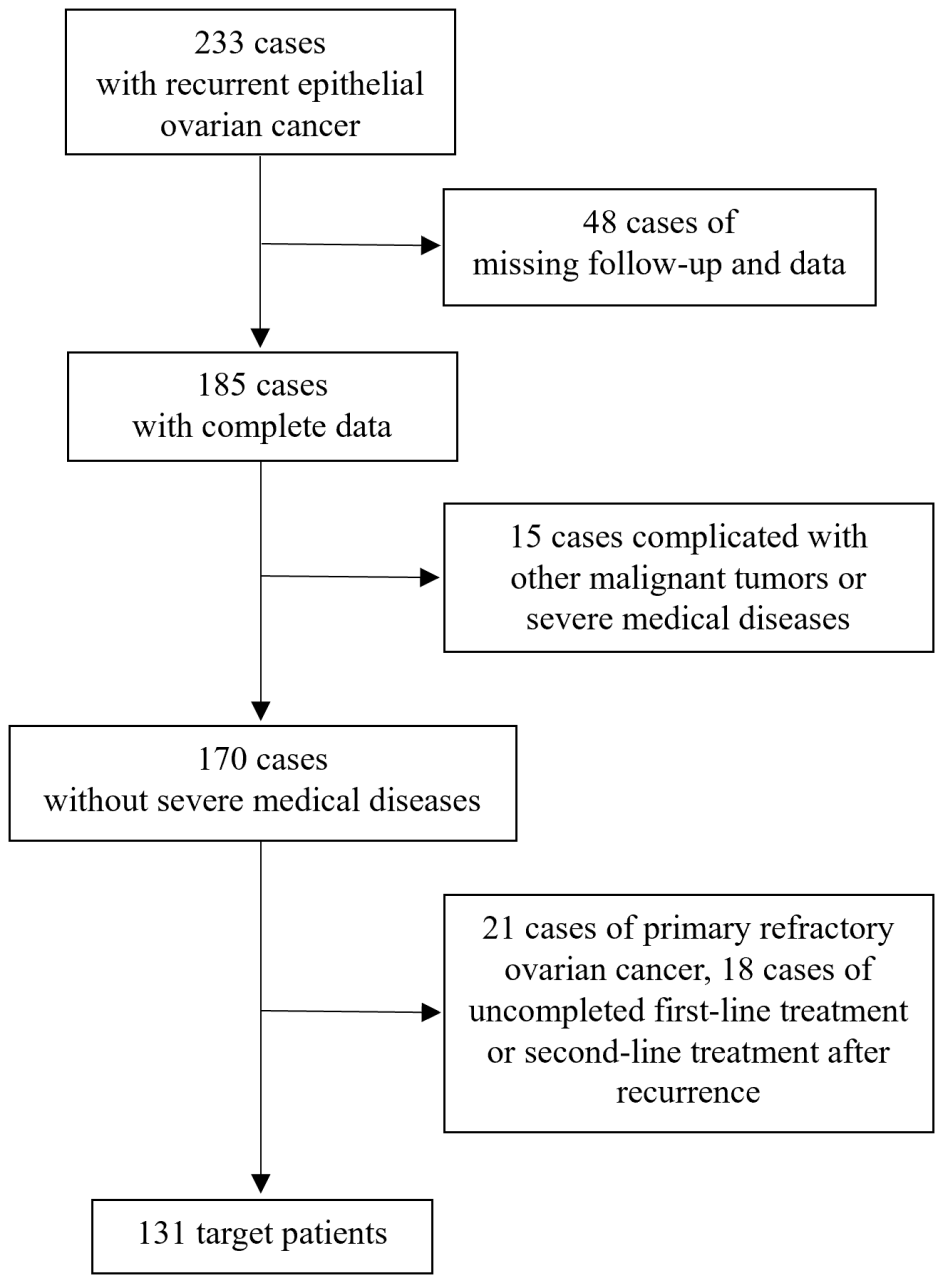
Patient screening flow chart.

As shown in **Figure 1**, 233 patients with recurrent epithelial ovarian cancer were treated at the First Affiliated Hospital of Wenzhou Medical University between December 2012 and December 2023. Among these, 48 patients were lost to follow-up or lack of clinical data, 15 patients had other malignant tumors or severe medical conditions, 21 patients had primary platinum-refractory ovarian cancer, 14 patients did not complete second-line treatment after recurrence, and 4 patients did not complete initial first-line treatment. Ultimately, 131 patients were included in the study.

### Comparison of baseline characteristics of patients with recurrent ovarian cancer

Among the 131 patients with recurrent ovarian cancer included in this study, 83 (63.4%) had received PARPis throughout their treatment, while 48 (36.6%) had not. Patients were divided into two cohorts: the non-PARPis and PARPis cohorts. Patient characteristics are detailed in **Table 1**. Significant differences were observed between the two groups in terms of BRCA gene status, neoadjuvant chemotherapy (NACT), postoperative residual condition, and platinum-free interval (PFI) after first-line treatment (*P* < 0.05).

**Table 1:** Comparison of baseline characteristics between two groups of patients with recurrent ovarian cancer.

	Non-PARPis-used cohort (N=48)	PARPis-used cohort (N=83)	*P* value	Overall population (N=131)
Age, years, x ± s	57.6 ± 10.9	57.8 ± 9.9	0.927	57.7 ± 10.2
BMI, kg/m^2^, x ± s	23.2 ± 3.2	22.8 ± 2.5	0.377	23.0 ± 2.8
CA125 level before initial treatment, kU/L, M(P_25_-P_75_)	904.8 (485.5-3432.1)	1402.1 (515.4-2842.8)	0.732	1262.7 (511.9-3070.1)
BRCA gene status, N (%)			<0.001	
BRCA wild type	1 (2.1)	44 (53.0)		45 (34.4)
BRCA mutant type	1 (2.1)	12 (14.5)		13 (9.9)
Not clear	46 (95.8)	27 (32.5)		73 (55.7)
FIGO staging, N (%)			0.050	
IA-IIA	0 (0.0)	5 (6.0)		5 (3.8)
IIB-IIIC	46 (95.8)	67 (80.7)		113 (86.3)
IVA-IVB	2 (4.2)	11 (13.3)		13 (9.9)
NACT, N (%)			0.012	
0	42 (87.5)	56 (67.5)		98 (74.8)
1	6 (12.5)	27 (32.5)		33 (25.2)
Postoperative residual disease status, N (%)			<0.001	
Optimal (R0/R1)	22 (45.8)	59 (71.1)		81 (61.8)
Suboptimal (≥R2)	26 (54.2)	19 (22.9)		45 (34.4)
Not clear	0 (0.0)	5 (6.0)		5 (3.8)
Pathological type, N (%)			0.076	
HGSOC	34 (70.8)	70 (84.3)		104 (79.4)
Others	14 (29.2)	13 (15.7)		27 (20.6)
First line chemotherapy combined with bevacizumab, N (%)			0.652	
0	47 (97.9)	79 (95.2)		126 (96.2)
1	1 (2.1)	4 (4.8)		5 (3.8)
First line chemotherapy course, N (%)			0.090	
≤ 6 courses	33 (68.8)	68 (81.9)		101 (77.1)
> 6 courses	15 (31.3)	15 (18.1)		30 (22.9)
Platinum containing chemotherapy efficacy, N (%)			0.606	
CR	40 (83.3)	74 (89.2)		114 (87.0)
PR	7 (14.6)	7 (8.4)		14 (10.7)
Not clear	1 (2.1)	2 (2.4)		3 (2.3)
PFI, N (%)			0.009	
≥1 month, <6 months	16 (33.3)	9 (10.8)		25 (19.1)
≥6 months, ≤12 months	12 (25.0)	27 (32.5)		39 (29.8)
>12 months	20 (41.7)	47 (56.6)		67 (51.1)
Only biochemical recurrence at the beginning of treatment, N (%)			0.355	
0	42 (87.5)	77 (92.8)		119 (90.8)
1	6 (12.5)	6 (7.2)		12 (9.2)
SCR, N (%)			0.839	
0	36 (75.0)	60 (72.3)		96 (73.3)
1	12 (25.0)	23 (27.7)		35 (26.7)
Second-line chemotherapy combined with bevacizumab, N (%)			1.000	
0	35 (72.9)	60 (72.3)		95 (72.5)
1	13 (27.1)	23 (27.7)		36 (27.5)

*PARPis*, PARP inhibitors; *BMI*, body mass index; *CA125*, carbohydrate antigen 125; *BRCA*, breast cancer 1; *FIGO*, The International Federation of Gynecology and Obstetrics; *NACT*, neoadjuvant chemotherapy; *HGSOC*, high-grade serous ovarian cancer; *CR*, complete remission; *PR*, partly remission; *PFI*, platinum free interval; *SCR*, secondary cytoreductive surgery.

### Comparison of the first-line use of PARPis on recurrence characteristics

As shown in **Table 2**, 27 patients (20.6%) received PARPis during first-line maintenance treatment, while 104 patients (79.4%) did not. Compared to those who did not receive PARPis in the first line, patients who maintained PARPis during first-line treatment exhibited no significant differences in distant metastasis, number of lesions, or CA125 levels at the recurrence time (*P* > 0.05). Both groups presented multiple lesions, distant metastasis, and elevated CA125 levels.

**Table 2:** Comparison of recurrence characteristics.

	Non-PARPis-used cohort (N=104)	PARPis-used cohort (N=27)	*P* value
Recurrence and distant metastasis, N (%)			0.949
1	48 (46.2)	14 (51.9)	
0	46 (44.2)	11 (40.7)	
Not clear	10 (9.6)	2 (7.4)	
Number of recurrent lesions, N (%)			0.301
1	34 (32.7)	5 (18.5)	
≥ 2	60 (57.7)	20 (74.1)	
Not clear	10 (9.6)	2 (7.4)	
CA125 level at recurrence, N (%)			0.797
> 35kU/L	81 (77.9)	22 (81.5)	
≤ 35kU/L	23 (22.1)	5 (18.5)	

### Survival analysis of patients with recurrent ovarian cancer

**Figure 2:**
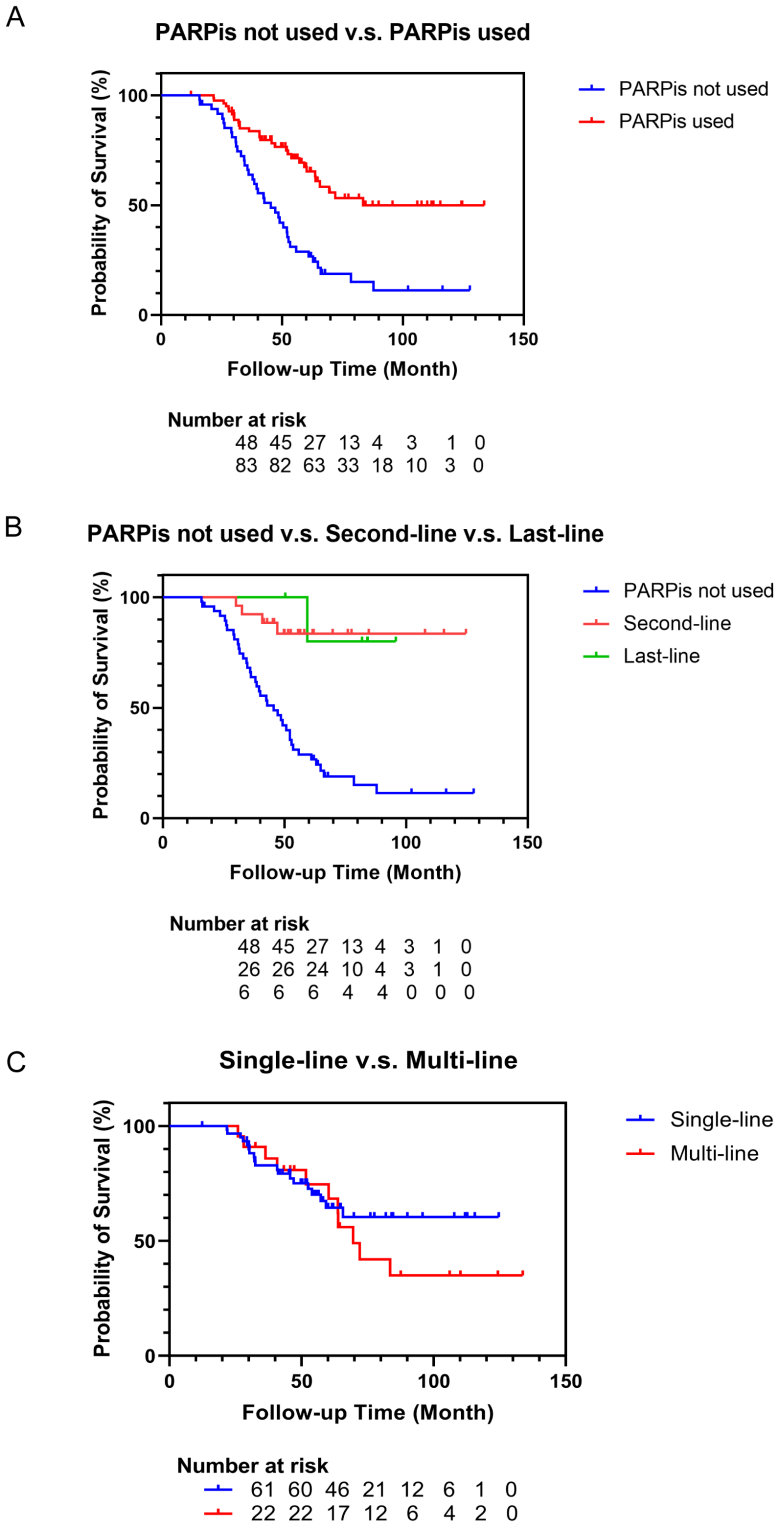
Survival curve of patients with recurrent ovarian cancer. **(A)** Survival curves of patients who not used or used PARPis as treatments. **(B)** Survival curves of patients who not used PARPis or used PARPis as the second-line or last-line treatments. **(C)** Survival curves of patients who used PARPis as the single-line or multi-line treatments.

All patients were followed up till December 2023. In the non-PARPis cohort, 39 patients (81.3%) died, whereas in the PARPis cohort, 30 patients (36.1%) died. The median follow-up time was 50.3 months (33.0-64.9 months). The median follow-up time for the non-PARPis cohort was 42.7 months (31.2-61.8 months), while for the PARPis cohort, it was 52.5 months (40.8-72.0 months). Kaplan-Meier survival curves were generated, and the log-rank test was used for comparisons. As shown in **Figure 2A**, the median OS of the non-PARPis cohort was 45.4 months (95% CI: 37.9-52.2 months). The median OS and 95% CI were not calculated for the PARPis cohort. Patients who received PARPis throughout their treatment had significantly better OS than those who did not (*P* < 0.0001).

In this study, various clinical data were collected and analyzed using univariate and multivariate Cox regression to assess the impact of clinical factors on OS in patients with recurrent ovarian cancer. As shown in **Table 3**, after adjusting for multiple factors, the use of PARPis throughout the treatment, CA125 levels before the first treatment, and the PFI after first-line treatment were identified as independent factors influencing OS in these patients (*P* < 0.05). Patients who received PARPis throughout their treatment, CA125 level ≤ 1000 kU/L before the first treatment and had a longer PFI exhibited better OS.

**Table 3:** Univariate and multivariate COX analysis of overall survival.

	Univariate COX analysis		Multivariate COX analysis	
	HR (95%CI)	*P* value	HR (95%CI)	*P* value
PARPis has been used in the whole treatment				
0	1		1	
1	0.334 (0.207-0.538)	<0.001	0.286 (0.142-0.575)	<0.001
Age				
< 60 years	1		–	–
≥ 60 years	1.191 (0.736-1.929)	0.476	–	–
CA125 level before initial treatment				
≤ 1000kU/L	1		1	
> 1000kU/L	2.282 (1.356-3.840)	0.002	2.027 (1.093-3.762)	0.025
FIGO stage				
IA-IIA	1	0.103	–	–
IIB-IIIC	4.076 (0.564-29.448)	0.164	–	–
IVA-IVB	7.173 (0.903-56.973)	0.062	–	–
NACT				
0	1		–	–
1	1.050 (0.618-1.784)	0.856	–	–
Postoperative residual disease status				
Optimal (R0/R1)	1		1	
Suboptimal (≥R2)	1.696 (1.029-2.797)	0.038	1.371 (0.683-2.751)	0.374
Pathological type				
HGSOC	1		–	–
Others	1.016 (0.573-1.802)	0.957	–	–
First line chemotherapy combined with bevacizumab				
0	1		1	
1	4.954 (1.486-16.515)	0.009	1.229 (0.306-4.945)	0.771
First line chemotherapy course				
≤ 6 courses	1		–	–
> 6 courses	1.405 (0.834-2.365)	0.201	–	–
Platinum containing chemotherapy efficacy				
CR	1		1	
PR	2.522 (1.183-5.379)	0.017	1.062 (0.422-2.672)	0.899
PFI				
≥1 month, <6 months	1	<0.001	1	<0.001
≥6 months, ≤12 months	0.360 (0.197-0.658)	0.001	0.421 (0.179-0.995)	0.049
>12 months	0.113 (0.061-0.211)	<0.001	0.116 (0.049-0.278)	<0.001
Recurrence and distant metastasis				
0	1		–	–
1	1.251 (0.758-2.065)	0.382	–	–
Number of recurrent lesions				
1			1	
≥ 2	2.381 (1.308-4.333)	0.005	1.812 (0.814-4.032)	0.145
CA125 level at recurrence				
> 35kU/L			1	
≤ 35kU/L	0.252 (0.109-0.583)	0.001	0.469 (0.159-1.377)	0.168
Only biochemical recurrence at the beginning of treatment				
0	1		–	–
1	1.148 (0.525-2.510)	0.730	–	–

As shown in **Table 4**, of the 83 patients who received PARPis throughout their treatment, 61 used PARPis for a single line during their overall management, while 22 used it in multiple lines. Among those who used PARPis for a single line, 51 patients used it as maintenance treatment and ten as salvage treatment. Specifically, 19 patients received PARPis during first-line treatment, 26 during second-line treatment, and six during last-line treatment.

**Table 4:** Different PARPis Treatment Patterns in patients with recurrent ovarian cancer.

Single-line				Multi-line
Maintenance Treatment			Salvage Treatment	
First-line	Second-line	Last-line	10	22
19	26	6		

In **Figure 2B**, Kaplan-Meier survival analysis was performed on the patients who used PARPis in the second-line and last-line maintenance therapy. The median OS and 95% CI for the second-line and last-line groups were not calculated. Compared with the patients who had not received PARPis treatment, the patients who used PARPis in the second-line (*P*<0.0001) and last-line (*P*=0.0063) maintenance treatment had better OS. There was no significant difference in OS between the second-line and last-line groups (*P*=0.8608). In **Figure 2C**, Kaplan-Meier survival analysis was conducted on patients who used PARPis repeatedly in multiple lines and those who used PARPis in only a single line. The median OS in the multi-line group was 69.5 months (95% CI: 60.3-83.5 months). The median OS and 95% CI for the single-line group were not calculated. There was no significant difference in OS between patients who used PARPis in multiple lines and those who used PARPis in only a single line (*P*=0.3919).

## Discussion

### Summary of main results

A total of 131 patients with recurrent ovarian cancer were included in this study. Of these, 83 patients (63.4%) received PARPis throughout their treatment, while 48 (36.6%) did not. Significant differences were observed between the two groups regarding BRCA gene status, NACT, postoperative residual condition, and PFI after first-line treatment (*P* < 0.05). As of the last follow-up, patients who received PARPis throughout their treatment had better OS than those who did not receive PARPis (*P* < 0.05). The use of PARPis throughout treatment, a CA125 level ≤ 1000 kU/L before the first treatment, and a longer PFI were identified as independent factors associated with improved OS in patients with recurrent ovarian cancer. First-line maintenance use of PARPis did not alter disease recurrence characteristics. PARPis administered in second-line and last-line treatments was associated with improved OS, although no significant difference in OS was found between these two stages of treatment. No evidence was found that patients who had previously used PARPis could derive additional OS benefits from reusing them again.

### Results in the context of published literature

This study demonstrated that disease progression patterns were similar in patients receiving either PARPis or placebo during first-line maintenance treatment. This finding aligns with the results of the SOLO-2 study presented at the ASCO meeting in 2022, which reported no significant difference in disease progression patterns between the PARPis and placebo groups (19). However, the SOLO-2 study focused on recurrence characteristics in patients with platinum-sensitive recurrent ovarian cancer who continued PARPis maintenance after recurrence. In contrast, our study examined patients with recurrent ovarian cancer more broadly. Additionally, SOLO-2 investigated a more comprehensive range of recurrence characteristics, including lesion sites categorized as target, non-target, and new lesions, and analyzed the distribution of specific progressive sites such as the liver, lung, and central nervous system.

The subgroup analysis in our study indicated that the use of PARPis as maintenance therapy, whether in second-line or last-line treatment, was associated with improved OS. However, no significant difference in OS was observed between these two groups. The L-MOCA subgroup analysis reported that the median progression-free survival (PFS) for patients receiving second-line treatment was 18.0 months, which was notably higher than that for patients receiving third-line treatment (≥third-line) (20). Although our study did not find a statistical difference in OS between the second-line and last-line treatment groups, the median OS for both groups had not been reached by the follow-up time, and the small sample size limits this conclusion.

Our study’s analysis of another subgroup did not find that multiple uses of PARPis improved OS compared to single-line use. The OReO study (21), a randomized, placebo-controlled Phase IIIB trial, included 220 patients with non-mucinous epithelial ovarian cancer who had previously received at least one line of PARPis maintenance treatment and had responded to the latest platinum-based chemotherapy. The study found better PFS through repeated maintenance treatment with Olaparib. Conversely, a real-world multi-center study in China (22) indicated no significant difference in PFS among patients with varying BRCA mutation statuses after receiving PARPis in a second time. This real-world study also included patients who received PARPis for salvage rather than maintenance. While our findings contrast with those of the OReO study, it is essential to note that our research focused on OS rather than PFS and involved a different patient population. In our study, some patients who received PARPis in multiple lines belonged to maintenance therapy, others had salvage therapy, and some had maintenance therapy followed by salvage therapy. Additionally, the control group of patients who received PARPis in a single line did not uniformly receive maintenance therapy.

### Strengths and weaknesses

This study divides recurrent ovarian cancer patients who have used PARPis into first-line, second-line, and last-line groups based on their medication courses and into single-line and multi-line groups based on their medication frequency. This is an innovative approach in retrospective researches. This study provides novel insights into the recurrence characteristics of ovarian cancer patients following first-line maintenance treatment with PARPis, an area with limited previous research. However, the study’s conclusions are constrained by its small sample size, being a single-center, retrospective analysis with only 27 patients who received PARPis during first-line maintenance. Further large-scale studies are needed to validate these findings. Additionally, the study’s limitations include the lack of BRCA gene detection data, which prevented an analysis of the benefit of BRCA mutations in patients receiving PARPis. The study also did not examine the recurrence characteristics of patients who maintained PARPis treatment after disease recurrence, representing another limitation.

### Implications for practice and future research

This study aids in classifying disease risk for patients, developing individualized follow-up plans, and demonstrating the potential survival benefits of PARPis for patients with recurrent ovarian cancer. It suggests that PARPis may extend OS in previously used patients. However, the study did not find a survival advantage from repeated use of PARPis, indicating that further exploration through large-scale randomized studies is needed in this area.

## Conclusion

Our results indicate significant differences in BRCA gene status, NACT, postoperative residual conditions, and PFI after first-line treatment between the non-PARPis and PARPis cohorts. The use of PARPis throughout the treatment course, a CA125 level ≤ 1000 kU/L before the first treatment, and a longer PFI were identified as independent factors associated with improved OS in patients with recurrent ovarian cancer. First-line maintenance with PARPis did not affect disease recurrence characteristics. While PARPis used as maintenance therapy during the whole line and in second-line and last-line treatments seemed to prolong OS according to our study, repeated use of PARPis did not confer additional OS benefits.

## Data Availability

The original contributions presented in the study are included in the article/supplementary material. Further inquiries can be directed to the corresponding authors.
